# Early Experience With Transcatheter Mitral Valve Replacement: A Systematic Review

**DOI:** 10.1161/JAHA.119.013332

**Published:** 2019-08-23

**Authors:** David del Val, Alfredo Nunes Ferreira‐Neto, Jerome Wintzer‐Wehekind, François Dagenais, Jean‐Michel Paradis, Mathieu Bernier, Kim O'Connor, Jonathan Beaudoin, Afonso B. Freitas‐Ferraz, Josep Rodés‐Cabau

**Affiliations:** ^1^ Quebec Heart & Lung Institute Laval University Quebec City Quebec Canada

**Keywords:** mitral valve disease, transcatheter cardiac therapeutics, transcatheter mitral valve replacement, Catheter-Based Coronary and Valvular Interventions

## Abstract

**Background:**

Transcatheter mitral valve replacement (TMVR) has emerged as an alternative therapeutic option for the treatment of severe mitral regurgitation in patients with prohibitive or high surgical risk. The aim of this systematic review is to evaluate the clinical procedural characteristics and outcomes associated with the early TMVR experience.

**Methods and Results:**

Published studies and international conference presentations reporting data on TMVR systems were identified. Only records including clinical characteristics, procedural results, and 30‐day and midterm outcomes were analyzed. A total of 16 publications describing 308 patients were analyzed. Most patients (65.9%) were men, with a mean age of 75 years (range: 69–81 years) and Society for Thoracic Surgery Predicted Risk of Mortality score of 7.7% (range: 6.1–8.6%). The etiology of mitral regurgitation was predominantly secondary or mixed (87.1%), and 81.5% of the patients were in New York Heart Association class III or IV. A transapical approach was used in 81.5% of patients, and overall technical success was high (91.7%). Postprocedural mean transmitral gradient was 3.5 mm Hg (range: 3–5.5 mm Hg), and only 4 cases (1.5%) presented residual moderate to severe mitral regurgitation. Procedural and all‐cause 30‐day mortality were 4.6% and 13.6%, respectively. Left ventricular outflow obstruction and conversion to open heart surgery were reported in 0.3% and 4% of patients, respectively. All‐cause and cardiovascular‐related mortality rates were 27.6% and 23.3%, respectively, after a mean follow‐up of 10 (range: 3 to 24) months.

**Conclusions:**

TMVR was a feasible, less invasive alternative for treating severe mitral regurgitation in patients with high or prohibitive surgical risk. TMVR was associated with a high rate of successful valve implantation and excellent hemodynamic results. However, periprocedural complications and all‐cause mortality were relatively high.

**Clinical Perspective**

**What Is New?**

- Early experience with transcatheter mitral valve replacement supports its feasibility, with a high rate of technically successful valve implantation in selected patients.
- Transcatheter mitral valve replacement shows excellent hemodynamic results with a low rate of residual mitral regurgitation and low transvalvular gradients.
- Procedure‐related complications and midterm mortality remain relatively high.

**What Are the Clinical Implications?**

- Transcatheter mitral valve replacement has emerged as an alternative treatment option for patients with prohibitive or high surgical risk.
- Further iterations of transcatheter mitral valve replacement systems, a gradual increase in use of the transfemoral–transeptal approach, and improved patient selection will be essential for a widespread adoption of this technology as an alternative to conventional surgery.

## Introduction

Mitral regurgitation (MR) is the leading cause of heart valve disease in Western countries and affects ≈10% of people aged >75 years.^1^ Conventional mitral valve surgery (either repair or replacement) remains the standard of care for patients with symptomatic severe MR, but close to half of such patients are not referred to conventional surgery because of potential comorbidities and high surgical risk.^2^ In recent years, several transcatheter mitral valve technologies have emerged as alternatives to surgery for the treatment of MR in patients deemed to be at increased surgical risk. Edge‐to‐edge leaflet repair (MitraClip; Abbott) is the transcatheter mitral valve repair (TMVr) system with the most experience and the only one approved by the US Food and Drug Administration to date. However, a percentage of patients remain suboptimal candidates for this technology, and residual moderate or severe MR after edge‐to‐edge leaflet repair has been reported in ≈10% of patients in real‐world practice.^3^, ^4^

Transcatheter mitral valve replacement (TMVR) has emerged as an alternative to TMVr for treating severe MR.^5^ A limited number of TMVR systems are under clinical evaluation, and others have started preclinical development programs. The objective of this systematic review is to evaluate the clinical and procedural characteristics and (early and midterm) clinical outcomes associated with TMVR.

## Methods

The data that support the findings of this study are available from the corresponding author on reasonable request.

A comprehensive systematic review of published data describing outcomes from different TMVR systems was performed in agreement with the guidance and the reporting items specified the Preferred Reported Items for Systematic Reviews and Meta‐Analysis (PRISMA) statement.^6^ A computerized search was performed to identify all relevant studies from the PubMed and Embase databases. Further data were sought by manual search of secondary sources, including references from primary papers (backward snowballing) and data reported in expert international conferences (Transcatheter Cardiovascular Therapeutics, Transcatheter Valve Therapies, Cardiovascular Research Technologies, EuroPCR, and PCR London Valves). The following keyword terms were used: *TMVR, TMVI* (transcatheter mitral valve implantation)*, transcatheter/percutaneous mitral valve replacement/implantation*. Databases were last accessed January 3, 2019, and studies were included if they were published in English. Transcatheter aortic valve systems used in patients with mitral surgery bioprosthetic or annuloplasty ring dysfunction (valve‐in‐valve and valve‐in‐ring procedures) or patients with severely mitral annular calcification (MAC) were beyond the focus of this systematic review.

The data were extracted using a standardized data abstraction sheet. Clinical characteristics, procedural results, and 30‐day and midterm outcomes were collected as reported by the authors. Two investigators (D.d.V. and A.N.F.‐N.) conducted the literature search selection and data extraction in duplicate. Any discrepancies were resolved by consensus, when needed, with a third investigator (J.R.‐C.). Gathered data included (when available) baseline clinical characteristics and relevant comorbidities, echocardiographic results, procedural details, procedure‐related complications, and follow‐up outcomes. Any TMVR system with at least 1 source (either published or reported in an international congress) describing a clinical experience was included. We excluded studies (1) that included patients using transcatheter aortic valve devices for valve‐in‐valve, valve‐in‐ring, or valve‐in‐MAC procedures; (2) that reported data from preclinical device evaluations; or (3) that reported results only on specialized news websites. When more than 1 reference provided data from the same cohort of patients, only the most recent or the one that provided more detailed data was included.

### Statistical Analysis

Continuous variables were expressed as mean±SD. Global cohort values were reported as weighted mean (range) or frequency (percentage). Weighted mean was calculated according to the total number of patients for each device (weight=n). When needed, the method devised by Hozo et al.^7^ was used for estimating the standard deviation of the sample. Data analyses were performed using the STATA software (v15.1; StataCorp).

## Results

**Figure 1:**
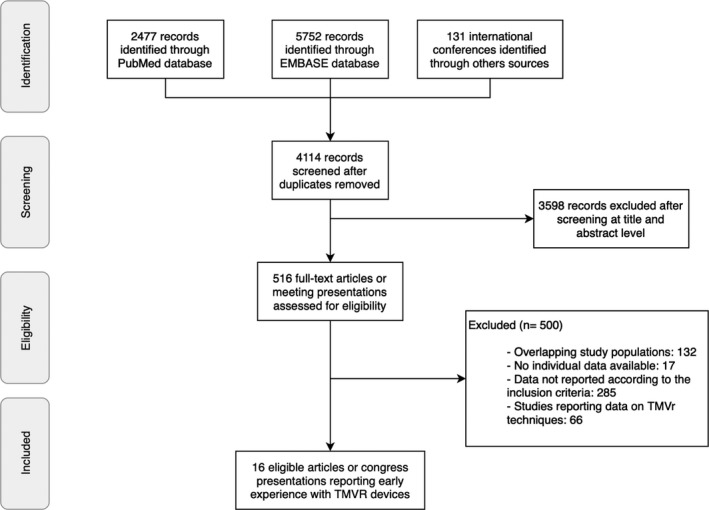
Flow diagram based on the Preferred Reported Items for Systematic Reviews and Meta‐Analysis (PRISMA) statement of studies and international conference presentations for evaluating early experience with TMVR. TMVr indicates transcatheter mitral valve repair; TMVR, transcatheter mitral valve replacement.

**Figure 2:**
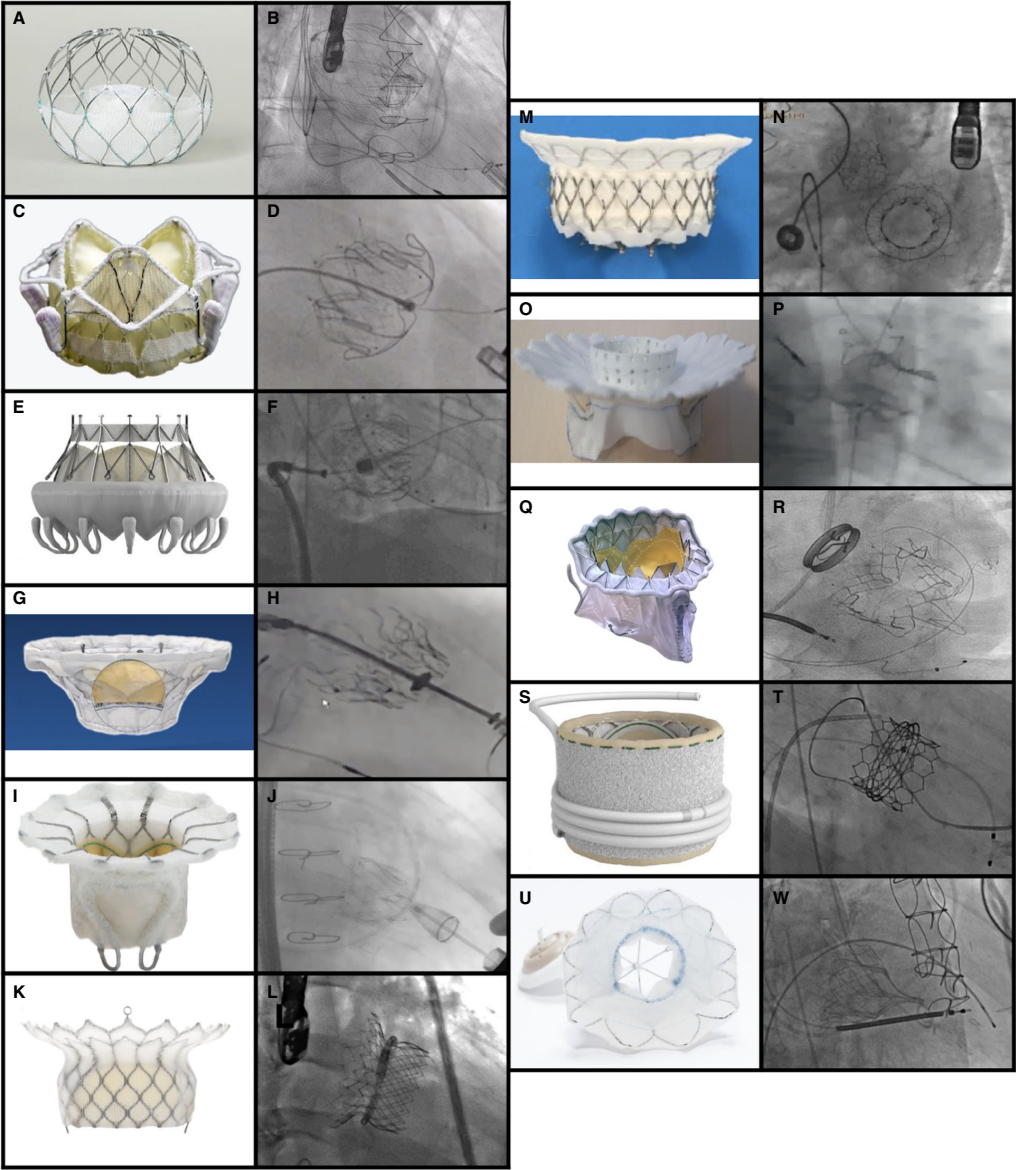
Transcatheter mitral valve replacement (TMVR) devices. **A**, AltaValve, provided by 4C Medical. **B**, Fluoroscopic image of the AltaValve. **C** and **D**, Provided by Caisson TMVR‐LivaNova: Caisson TMVR (**C**); fluoroscopy image of the Caisson TMVR (**D**). **E**, CardiAQ Valve, provided by Edwards Lifesciences. **F**, Fluoroscopy image of the CardiAQ Valve. **G** and **H**, Courtesy of Dr Francesco Maisano, Heart Center University Hospital, Zurich, Switzerland: CardioValve (**G**); fluoroscopy image of the CardioValve (**H**). **I** and **J**, Reprinted from Regueiro et al^15^ with permission from Elsevier: Fortis (**I**); fluoroscopy image of the Fortis (**J**). **K** and **L**, Courtesy of HighLife Medical: HighLife (**K**); fluoroscopy image of the HighLife (**L**). **M** and **N**, Courtesy of Dr Vinayak Bapat, New York Presbyterian Hospital/Columbia University Medical Center: Intrepid TMVR (**M**); fluoroscopy image of the Intrepid TMVR (**N**). **O**, MValve System. **P**, Fluoroscopy image of the MValve System, courtesy of Dr Maurice Buchbinder, Stanford University/VA Palo Alto Healthcare System, California. **Q** and **R**, Provided by Neovasc Medical Inc: Tiara (**Q**); fluoroscopy image of the Tiara (**R**). **S** and **T**, Courtesy of Dr John Webb, St. Paul's Hospital, Vancouver, Canada: Sapien M3 System (**S**); fluoroscopy image of the Sapien M3 System (**T**). **U**, Tendyne. **W**, Fluoroscopy image of the Tendyne.

The searches of PubMed, Embase, and the main international conferences identified 2477, 5752, and 131 records, respectively, yielding 4114 records that were reviewed at the title and abstract level after exclusion of duplicates. Of those, 516 were selected and assessed for eligibility. Finally, 16 publications or conference presentations describing the experience with 11 different dedicated TMVR systems in 308 patients were included.^8^, ^9^, ^10^, ^11^, ^12^, ^13^, ^14^, ^15^, ^16^, ^17^, ^18^, ^19^, ^20^, ^21^, ^22^, ^23^ Figure 1 shows the PRISMA flow diagram. The main features of all TMVR systems (Figure 2) have been described in detail previously and are summarized in Table 1.^5^, ^8^, ^24^, ^25^, ^26^, ^27^, ^28^, ^29^

**Table 1:** Characteristics of Main TMVR Devices

Device	Manufacturer	Frame	Leaflets	Anchoring Mechanism	Approach and Delivery System Diameter	Valve Size (mm)
AltaValve	4C Medical Technologies	Self‐expanding, nitinol	3 bovine leaflets	Spherical frame shape	Transapical 32‐Fr	27
Caisson TMVR	LivaNova	Self‐expanding, nitinol	3 porcine leaflets	4 subannular anchoring feet 3 atrial holding features	Transfemoral–transeptal 31‐Fr	36A 42A 42B
CardiAQ Valve	Edwards Lifesciences	Self‐expanding, nitinol	3 bovine leaflets	LV anchors that engage the native mitral leaflets and annulus	Transapical Transfemoral–transeptal 33‐Fr	30
CardioValve	Cardiovalve				Transfemoral‐transeptal 28‐Fr	3 sizes
Fortis	Edwards Lifesciences	Cloth‐covered, self‐expanding, nitinol	3 bovine leaflets	2 Opposing paddles	Transapical 42‐Fr	29
HighLife	HighLife SAS	Self‐expanding, nitinol	3 bovine leaflets	Valve in subannular mitral ring; external anchor	Transapical (transfemoral artery for loop placement) 39‐Fr	31
Intrepid TMVR	Medtronic	Double stent, self‐expanding, nitinol	3 bovine leaflets	Radial force and small cleats on outer stent engage leaflets	Transapical 35‐Fr	27 (with 3 outer stent sizes: 43, 46 and 50 mm)
MValve system	MValve Technologies	Dock system to be used with commercially available valves	NA	External anchor	Transapical 32‐Fr	NA
Tiara	Neovasc	Self‐expanding, nitinol	3 bovine leaflets	3 ventricular anchoring tabs (onto the fibrous trigone and posterior shelf of the annulus)	Transapical 32‐Fr (35‐mm valve) 36‐Fr (40‐mm valve)	35 and 40
Sapien M3	Edwards Lifesciences	Balloon‐expandable, cobalt‐chromium frame	3 bovine leaflets	Nitinol dock system	Transfemoral 20‐Fr	29
Tendyne	Abbott	Double frame, self‐expandable, nitinol	3 porcine leaflets	Apical tether	Transapical 34‐Fr	Outer (sealing) frame ranges 30–43 mm in the SL dimension and 34–50 in the IC dimension

IC indicates intercommisural; LV, left ventricular; NA, not available; SL, septal‐to‐lateral; TMVR, transcatheter mitral valve replacement.

### Baseline Characteristics

The main baseline clinical characteristics of TMVR recipients are described in Table 2. The mean age of the patients was 75 years (range: 69–81 years), and 65.9% of patients were men. Most patients (81.5%) were in New York Heart Association (NYHA) class III or IV, and almost half (47.2%) exhibited at least 1 episode of heart failure hospitalization within the year before the TMVR procedure. The mean Society for Thoracic Surgery Predicted Risk of Mortality (STS‐PROM) score was 7.7±0.8%, ranging from 6.14% to 8.6%. Severe comorbidities were frequent, including coronary artery disease (70.3%), prior coronary artery bypass grafting (44.2%), chronic renal insufficiency (60.3%), and atrial fibrillation (55.2%). Mean left ventricular (LV) ejection fraction was 42±4% (range: 30–46%). The indication for TMVR was predominantly secondary (functional) or mixed severe MR (87.1%), and 36 patients (12.9%) were diagnosed with primary MR.

**Table 2:** Baseline Clinical Characteristics

	AltaValve (n=1)	Caisson (n=23)	CardiAQ (n=26)	Cardio‐ Valve (n=5)	Fortis (n=13)	HighLife (n=15)	Intrepid (n=50)	MValve System (n=1)	Tiara (n=59)	Sapien M3 (n=15)	Tendyne (n=100)	Global Cohort (N=308)^a^
Age, y	77	81	80±8	NA	71±8	69 (50–79)	NA	75	74±10	76 ±12	75.4 ±8.1	75.2 (69–81)
Female	0 (0)	13/20 (65)	2/14 (14.3)	NA	3 (23.1)	3 (20)	21 (42)	NA	16 (27)	9 (60)	31 (31)	98/287 (34.1)
STS‐PROM Score (%)	NA	8.3 ±3.3	6.14	NA	7.2±3.6	NA	6.4±5.5	NA	8.6±7.1	8.3±4.3	7.8 ±5.7	7.7 (6.1–8.6)
Etiology of MR												
Primary		4/20 (20)	6 (23.1)	NA	0 (0.0)		8 (16.0)	1 (100)	6/58 (10)	NA	11 (11)	36/280 (12.9)
Secondary	1 (100)	11/20 (55)	20 (76.9)	NA	12 (92.3)	11 (73)	36 (72.0)		37/58 (64)	NA	89 (89)	217/280 (77.5)
Mixed MR		5/20 (25)		NA	1 (7.7)		6 (12.0)		15/58 (26)	NA		27/280 (9.6)
Grade III or IV MR severity	NA	23 (100)	12/12 (100)	NA	13 (100)	NA	49 (98)	NA	NA	15 (100)	99 (99)	211/213 (99.1)
NYHA class												
III–IV	1 (100)	16/20 (70)	NA	NA	13 (100)		43 (86)	1 (100)	56 (95)	15 (100)	66 (66)	211/259 (81.5)
LV ejection fraction	30	45.0±11.5	42.88	NA	34±9	38 (27–54)	43.4±11.8	NA	37.2±8	39.8 ±9.4	46.4 ±9.6	42.4 (30–46.4)
Diabetes mellitus	NA	6 (25)	0/4 (0)	NA	5 (38.5)	NA	21 (42.0)	NA	NA	NA	38 (38)	70/190 (36.8)
Hypertension	NA	22 (95)	NA	NA	NA	NA	NA	NA	NA	NA	80 (80)	102/123 (82.9)
Atrial fibrillation	1 (100)	13 (55)	2/4 (50)	NA	8 (61.5)	NA	29 (58.0)	NA	33 (56)	5 (33)	NA	91/165 (55.2)
Coronary artery disease	1 (100)	16 (70)	NA	NA	10 (76.9)	NA	34 (68.0)	NA	NA	7 (46.7)	74 (74)	142/202 (70.3)
Prior myocardial infarction	NA	7 (30)	3/4 (75)	NA	NA	NA	22 (44.0)	NA	NA	NA	57 (57)	89/177 (50.3)
Prior coronary artery bypass surgery	1 (100)	NA	10/13 (77)	NA	7 (53.8)	NA	19 (38.0)	NA	21 (36)	6 (40)	47 (47)	111/251 (44.2)
Prior valve intervention/surgery	1 (100)	15 (65)	NA	NA	NA	5 (33)	5 (10.0)	1 (100)	15 (25)	NA	0 (0)	42/249 (16.9)
Chronic renal insufficiency	NA	10 (45)	NA	NA	NA	NA	29 (58.0)	NA	45 (76)	5 (33)	60 (60)	149/247 (60.3)
Chronic obstructive pulmonary disease	NA	8 (35)	2/4 (50)	NA	5 (38.5)	NA	25 (50.0)	NA	16 (27)	NA	39 (39)	95/249 (38.2)
Prior stroke or TIA	NA	2 (10)	1/4 (25)	NA	3 (23.1)	NA	8 (16.0)	NA	NA	NA	16 (16)	30/190 (15.8)
Pulmonary hypertension	NA	9 (40)	3/7 (43)	NA	NA	NA	20 (40.0)	NA	NA	NA	NA	32/80 (40.0)
Hospitalization for HF within past year	NA	NA	NA	NA	9 (69.2)	NA	29 (58.0)	NA	NA	NA	39 (39)	77/163 (47.2)

Values are mean±SD or n (%) except as noted. HF indicates heart failure; LV, left ventricular; MR, mitral regurgitation; NA, not available; NYHA, New York Heart Association; STS‐PROM, Society of Thoracic Surgeons Predicted Risk of Mortality; TIA, transient ischemic attack.

a Values are weighted mean (range) or n/N (%).

### Procedural and 30‐Day Results

Procedural details and 30‐day outcomes are shown in Table 3. The vast majority of procedures were performed under general anesthesia and transesophageal echocardiographic guidance. Most patients (81.5%) underwent TMVR through the transapical approach. The valve was successfully implanted in most patients, with overall reported technical success of 91.7%. Early TMVR‐related complications included valve malposition in 8 of 236 patients (3.4%), device embolization or migration in 2 of 254 patients (0.8%), valve thrombosis in 3 of 179 patients (1.7%), and LV outflow tract obstruction and acute valve dysfunction in 1 patient each (0.4%). These complications led to conversion to open heart surgery in 11 of 272 patients (4.0%). Procedure‐related mortality was reported in 13 of 280 patients (4.6%). Data on residual MR were available for most patients, with only 4 (1.5%) presenting moderate to severe MR after the procedure. Postprocedural mean transmitral gradient was 3.5mm Hg (range: 3.0–5.53 mm Hg). Patients were discharged at a mean of 10 days (range: 6.3–11.1 days) after the procedure. All‐cause 30‐day mortality and rehospitalization rates were 13.6% (range: 0–60%) and 15.8% (12 of 76 patients), respectively.

**Table 3:** Procedural and 30‐Day Clinical Outcomes

	AltaValve	Caisson	CardiAQ	CardioValve	Fortis^a^	HighLife	Intrepid	MValve System	Tiara	Sapien M3	Tendyne	Global Cohort^b^
All‐cause 30‐d mortality	0/1 (0.0)	2/11 (18.2)	7/26 (26.9)	3/5 (60.0)	5/13 (38.5)	3/15 (20.0)	7/50 (14.0)	1/1 (100)	6/58 (10.3)	0/15 (0.0)	6/100 (6.0)	40/295 (13.6)
Procedure‐related mortality	0/1 (0.0)	NA	3/26 (11.5)	1/5 (20.0)	4/13 (30.8)	2/11 (18.2)	3/50 (6.0)	NA	0/59 (0.0)	0/15 (0.0)	0/100 (0.0)	13/280 (4.6)
Technical success	1/1 (100)	18/23 (78.3)	22/26 (84.6)	5/5 (100)	10/13 (76.9)	8/11 (72.7)	48/49 (97.9)	1/1 (100)	56/59 (94.9)	13/15 (86.7)	97/100 (97.0)	278/303 (91.7)
Procedure time, min	NA	177 ±65	NA	43.6±13.1	123±27	NA	100 (80–124)	NA	82 (60–155)	189 ±100	136.1±36.3	121.4 (43.6–189)
Approach												
Transfemoral		23/23 (100)	14/26 (53.8)	5/5 (100)						15/15 (100)		57/308 (18.5)
Transapical	1/1 (100)		12/26 (46.2)		13/13 (100)	15/15 (100)	50/50 (100)	1/1 (100)	59/59 (100)		100/100 (100)	251/308 (81.5)
Conversion to open heart surgery	0/1 (0.0)	4/23 (17.4)	NA	NA	2/13 (15.4)	2/11 (18.2)	0/50 (0.0)	NA	3/59 (5.1)	0/15 (0.0)	0/100 (0.0)	11/272 (4.0)
LVOT obstruction	0/1 (0.0)	0/17 (0.0)	NA	0/5 (0.0)	0/13 (0.0)	1/15 (6.6)	0/50 (0.0)	NA	0/58 (0.0)	0/15 (0.0)	0/100 (0.0)	1/274 (0.4)
Device embolization or migration	0/1 (0.0)	0/17 (0.0)	NA	NA	0/13 (0.0)	NA	0/50 (0.0)	NA	2/58 (3.4)	0/15 (0.0)	0/100 (0.0)	2/254 (0.8)
Malposition	0/1 (0.0)	NA	1/13 (7.7)	NA	1/13 (7.7)	NA	1/50 (2.0)	NA	3/59 (5.1)	NA	2/100 (2.0)	8/236 (3.4)
Moderate or severe MR	0/1 (0.0)	1/11 (9.1)	0/26 (0.0)	0/5 (0.0)	0/13 (0.0)	0/15 (0.0)	0/50 (0.0)	NA	1/37 (2.7)	1/15 (6.6)	1/100 (1.0)	4/273 (1.5)
Valve dysfunction	0/1 (0.0)	NA	NA	NA	0/13 (0.0)	0/11 (0.0)	0/50 (0.0)	NA	NA	1/15 (6.6)	0/100 (0.0)	1/190 (0.5)
Device thrombosis	0/1 (0.0)	NA	NA	NA	1/13 (7.7)	1/15 (6.6)	0/50 (0.0)	NA	NA	NA	1/100 (1.0)	3/179 (1.7)
Stroke	0/1 (0.0)	0/11 (0.0)	NA	NA	0/13 (0.0)	0/15 (0.0)	2/50 (4.0)	NA	2/37 (5.4)	1/15 (6.6)	2/100 (2.0)	7/242 (2.9)
Bleeding	0/1 (0.0)	0/11 (0.0)	1/13 (7.7)	1/5 (20.0)	0/13 (0.0)	NA	9/50 (18.0)	NA	NA	0/15 (0.0)	20/100 (20.0)	31/208 (14.9)
Access site complication	0/1 (0.0)	0/11 (0.0)	NA	1/5 (20.0)	NA	NA	0/50 (0.0)	NA	4/58 (6.9)	0/15 (0.0)	1/100 (1.0)	6/240 (2.5)
Acute kidney injury	0/1 (0.0)	NA	NA	NA	2/13 (15.4)	NA	5/50 (10.0)	NA	12/37 (32.4)	1/15 (6.6)	8/100 (8.0)	28/216 (13.0)
Mean transmitral gradient, mm Hg	NA	3.1	NA	4.4	3±1	NA	4.1±1.3	NA	NA	5.53±2.2	3.0±1.1	3.5 (3.0–5.53)
Length of stay, d	9	NA	NA	NA	10±6	NA	NA	NA	NA	6.3 ±3.2	11.1±8.7	10.4 (6.3–11.1)

Values are mean±SD or n/N (%) except as noted. LVOT indicates left ventricular outflow tract; MR, mitral regurgitation; NA, not available.

a In late 2015, Edwards Lifesciences stopped the Fortis program. The valve is not currently available.

b Values are weighted mean (range) or n/N (%).

### Midterm Follow‐Up

Follow‐up (>30‐day) data were available for 7 of 11 TMVR systems. Mean follow‐up was 10.1±0.3 months, ranging from 3 to 24 months. The main clinical outcomes during the follow‐up period are summarized in Table 4. The all‐cause mortality rate was 27.6% (71/257 patients), with cardiovascular‐related mortality of 23.3% (38/163 patients). Functional status improved in the majority of patients, with 125 of 146 (85.6%) exhibiting NYHA class I or II at follow‐up. Overall, 26.4% of patients were hospitalized because of acute decompensated heart failure during the follow‐up period, and a stroke event occurred in 8 of 176 patients (4.5%). Echocardiographic data at follow‐up were available for 83 patients, with no cases of moderate to severe MR and a mean transmitral gradient of 3.3±0.3 mm Hg. TMVR‐related complications including device thrombosis, device hemolysis, and endocarditis were reported in 3 of 100 (3.0%), 6 of 150 (4.0%), and 4 of 150 (4.0%) patients, respectively. Reintervention for mitral valve dysfunction was required in 4 of 174 patients (2.3%). There were no cases of device fracture or embolization during the follow‐up period.

**Table 4:** Midterm Clinical Outcomes

	Caisson	CardiAQ	Fortis^a^	HighLife	Intrepid	Tiara	Tendyne	Global Cohort^b^
Follow‐up, mo	9.9	NA	24^a^	12	7.04±6.7	3	13.7	10.1 (3–24)
Any mortality	2/11 (18.2)	9/13 (69.2)	7/13 (53.8)	4/15 (26.7)	11/50 (22.0)	12/55 (21.8)	26/100 (26)	71/257 (27.6)
Cardiovascular mortality	NA	NA	5/13 (38.5)	NA	11/50 (22.0)	NA	22/100 (22)	38/163 (23.3)
NYHA class III–IV	1/9 (11.1)	NA	1/8 (12.5)	NA	9/43 (20.9)	1.9±0.6	10/86 (11.6)	21/146 (14.4)
Mean transmitral gradient, mm Hg	NA	NA	3±1	NA	4.1±1.3	NA	3.0±1.1	3.3 (3–4.1)
Moderate or severe MR	0/9 (0.0)	0/12 (0.0)	0/8 (0.0)	0/12 (0.0)	0/42 (0)	NA	NA	0/83 (0.0)
Stroke	2/11 (18.2)	NA	NA	0/15 (0.0)	3/50 (6.0)	NA	3/100 (3)	8/176 (4.5)
Myocardial infarction	0/11 (0.0)	NA	NA	0/15 (0.0)	0/50 (0.0)	NA	4/100 (4)	4/176 (2.3)
HF hospitalization	1/11 (9.1)	NA	2/13 (15.4)	NA	12/50 (24.0)	NA	31/100 (31)	46/174 (26.4)
Reintervention for mitral valve	0/11 (0.0)	NA	0/13 (0.0)	NA	0/50 (0.0)	NA	4/100 (4)	4/174 (2.3)
Bioprosthetic valve dysfunction	NA	NA	0/13 (0.0)	0/15 (0.0)	NA	NA	0/100 (0)	0/128 (0.0)
Device hemolysis	NA	NA	NA	NA	NA	NA	3/100 (3)	3/100 (3.0)
Device embolization	NA	NA	0/13 (0.0)	NA	0/50 (0.0)	NA	0/100 (0)	0/163 (0.0)
Device thrombosis	NA	NA	NA	NA	0/50 (0.0)	NA	6/100 (6)	6/150 (4.0)
Fracture	NA	NA	0/13 (0.0)	NA	NA	NA	0/100 (0)	0/113 (0.0)
Endocarditis	NA	NA	NA	NA	2/50 (4.0)	NA	2/100 (2)	4/150 (2.7)

Values are mean±SD or n/N (%) except as noted. HF indicates heart failure; MR, mitral regurgitation; NA, not available; NYHA, New York Heart Association.

a In late 2015, Edwards Lifesciences stopped the Fortis program. The valve is not currently available.

b Values are weighted mean (range) or n/N (%).

## Discussion

The main findings of this systematic review on the initial TMVR experience in patients with severe MR can be summarized as follows. First, the data support the feasibility of TMVR, with a high rate of successful valve implantation. Second, TMVR was associated with excellent hemodynamic results, with a very low rate of significant residual MR and low transvalvular mitral gradients after the procedure. Third, the rates of periprocedural complications and early mortality were relatively high. Fourth, most patients improved their functional class, but the mortality rate remained high (28%) after a mean follow‐up of about 1 year.

### Patient Selection Considerations

This review showed that patients selected for TMVR were elderly and exhibited a high comorbidity burden, leading to high to prohibitive surgical risk. In addition, most patients had secondary MR, with reduced LV ejection fraction. Appropriate patient selection for TMVR may be challenging and requires a global assessment including patient comorbidities, mechanisms of MR, and structural/anatomical features. Unfortunately, limited data were provided on inclusion and exclusion criteria and screen failure rates in most TMVR series. Although these details were formally reported in only 2 registries, the global rate of screen failure was presumably high. In the Tendyne Global Feasibility Study, only 100 of 332 patients screened were finally included (screen failure ≈70%).^23^ Similarly, in the Global Pilot Study with the Intrepid TMVR system, only 50 of 166 patients screened were selected. The most common exclusion criteria were severe LV dysfunction, large annulus dimensions, high risk of LV outflow tract obstruction, severe mitral annular or leaflet calcification, previous mitral or aortic valve surgery, intracardiac thrombus, severe pulmonary hypertension, severe tricuspid valve regurgitation, and severe right ventricular dysfunction. Moreover, patient selection was limited by valve size availability in some cases.

The mitral valve apparatus is a complex dynamic structure and multiple important anatomical aspects should be considered. Unlike transcatheter aortic valve replacement, TMVR must address challenges such as (1) noncircular saddle‐shape dynamic annulus of large dimensions, (2) lack of calcified or rigid mitral annulus, (3) irregular leaflets geometry, (4) proximity of LV outflow tract, and (5) the presence of the subvalvular apparatus. Thus, the use of imaging techniques like 3‐dimensional computed tomography is key for evaluating patient eligibility, and anatomical issues remain the most important factor determining TMVR feasibility.^30^

### Procedural Aspects and Early Outcomes

The vast majority of procedures (>80%) were performed through a transapical approach, with only 4 devices implanted through a transfemoral–transeptal approach Sapien M3 (Edwards Lifesciences, Irvine, CA, USA), Caisson (LivaNova PLC, London, UK), CardiAQ (Edwards Lifesciences, Irvine, CA, USA) and CardioValve (Cardiovalve Ltd., Or Yehuda, Israel). This choice is related to the large size of the valve‐delivery catheters along with the location of the mitral valve, which makes the appropriate alignment of the transcatheter valve more difficult when using the antegrade transeptal approach. Although this study represented the initial experience with different TMVR platforms, technical success was high, and successful valve implantation was achieved in up to 92% of cases (range: 73–100%). In addition, significant LV outflow tract obstruction was reported in only 1 case, probably reflecting the appropriateness of preprocedural imaging workup along with conservative eligibility criteria. However, conversion to open heart surgery was required in 4% of patients. The causes were chordal entanglement (2 cases), valve malposition (4 cases), and the presence of multiple papillary muscles, a septal bulge, and a thickened LV posterior wall impeding the proper placement of a wire after apical puncture (1 case). While these complications may be part of the learning curve process, it highlights the importance of heart teams and specialized centers with high surgical experience dedicated to these procedures.

Importantly, the results regarding valve performance showed a very low rate of significant residual MR (moderate or severe: 1.5%) and low transvalvular gradients (mean: <4 mm Hg) following the procedure. These results appear to be similar to those associated with surgical mitral valve repair and replacement^3^ and superior to those reported in transcatheter TMVr series, including the most recent studies with the MitraClip device.^31^, ^32^, ^33^, ^34^, ^35^ Despite the good results regarding procedural success and valve performance, the 30‐day mortality rate after TMVR was as high as 13.6% (varying from 0% to 60%), which exceeded the estimated surgical risk (mean STS‐PROM of the study population: ≈8%) and was much higher than that reported in TMVr series including patients with a similar risk profile.^31^, ^36^ The causes of such high periprocedural mortality are probably multifactorial. First, in addition to the significant comorbidity burden, the fact that most patients had secondary MR as the underlying disease may have contributed.^37^, ^38^ Second, the rate of periprocedural complications like major or life‐threatening bleeding or acute kidney injury—both associated with poorer outcomes—was high (>10%). This may be partially related to the use of the transapical approach in most patients, which has also been associated per se with a negative clinical impact, particularly in elderly and frail patients.^39^, ^40^ Third, the myocardial injury secondary to the transapical approach may be particularly deleterious in patients with already reduced ventricular function.^41^ Similar to the transcatheter aortic valve replacement field, a progressive transition toward increasing use of transfemoral systems will likely occur in the near future and may translate into improved outcomes. Finally, the learning curve process probably contributed to the high mortality rate observed in this initial TMVR experience.

### Midterm Outcomes

Although the results were highly heterogeneous among different TMVR systems, the mean mortality rate was as high as 28% after a mean follow‐up close to 1 year. This rate is similar to that reported by the STS/ACC TVT Registry (Society of Thoracic Surgeons/American College of Cardiology Transcatheter Valve Therapy) including 2952 patients (mean STS‐PROM: ≈6%) with secondary MR treated with the MitraClip system.^31^ Although the high noncardiac comorbidity burden of TMVR recipients may have contributed, it has to be noted that about half of the deaths were related to cardiovascular causes. In addition, a significant proportion of patients were hospitalized for heart failure decompensation within the months following the procedure. Importantly, no deaths specifically related to transcatheter valve dysfunction were reported. Two recent trials evaluating the efficacy of TMVr with the MitraClip system for treating secondary MR provided contradictory results,^34^, ^35^ highlighting the importance of adequate patient selection to avoid treatment futility in this population. Future studies are needed to determine which patients may benefit the most from TMVR.

Although the follow‐up duration was limited, no signs of early valve degeneration were detected following TMVR (up to 1‐year follow‐up). Future studies with longer follow‐up should provide extended valve durability data. However, device thrombosis was reported in 3% of patients, highlighting the importance of appropriate antithrombotic treatment following these procedures. Anticoagulation therapy for a minimum of 3 months was recommended in most TMVR studies, probably mimicking the recommendations from current guidelines regarding conventional surgical mitral valve replacement. Future studies will need to evaluate the optimal antithrombotic strategy in these patients.

### TMVR Versus TMVr

Several TMVr techniques, based on surgical repair techniques, are being simultaneously developed as a minimally invasive alternative to patients who are not candidates for surgery. MITRA‐FR (Multicentre Study of Percutaneous Mitral Valve Repair MitraClip Device in Patients With Severe Secondary Mitral Regurgitation) showed no benefit of MitraClip in patients with severe secondary MR on optimized medical treatment.^35^ However, the COAPT (Clinical Outcomes Assessment of the MitraClip Percutaneous Therapy) trial demonstrated significantly lower all‐cause mortality and rehospitalization for heart failure in patients who underwent the MitraClip procedure.^34^ Main patient baseline characteristic and results comparing the TMVR population with the COAPT device group are summarized in Table 5.

**Table 5:** Baseline Characteristics, Procedural and 30‐Day Results, and Midterm Outcomes Comparing the TMVR Cohort With the COAPT Device Group

	TMVR (N=308)	COAPT Device Group (N=302)
Characteristics of the patients at baseline		
Age, y	75.2±3.5	72.2±11.2
STS‐PROM score, %	7.7±0.75	7.8±5.5
Secondary MR	217/280 (77.5)	302/302 (100)
Grade III or IV MR severity	211/213 (99.1)	302/302 (100)
Left ventricular ejection fraction	42.4±4.7	31.3±9.3
Hypertension	102/123 (82.9)	243/302 (80.5)
Diabetes mellitus	70/190 (36.8)	106/302 (35.1)
Prior myocardial infarction	89/177 (50.3)	156/302 (51.7)
Prior coronary artery bypass surgery	111/251 (44.2)	121/302 (40.1)
Chronic obstructive pulmonary disease	95/249 (38.2)	71/302 (23.5)
Hospitalization for HF within past year	77/163 (47.2)	176/302 (58.3)
Procedural and 30‐d clinical outcomes		
Procedure time, min	121.4±41.9	162.9±118.1
All‐cause 30‐d mortality	40/295 (13.6)	7/302 (2.3)
Stroke	7/242 (2.9)	2/302 (0.7)
Moderate or severe MR	4/273 (1.5)	20/273 (7.3)
Unplanned mitral valve surgery	11/272 (4.0)	0/302 (0.0)
Midterm clinical outcomes^a^		
Any mortality during follow‐up	71/257 (27.6)	57/302 (18.9)
NYHA class III–IV	21/146 (14.4)	48/237 (20.3)
Moderate or severe MR	0/83 (0.0)	11/210 (5.2)
HF hospitalization	46/174 (26.4)	92/302 (35.7)
Unplanned mitral valve reintervention	4/174 (2.3)	10/302 (3.3)^b^
Device embolization or migration	2/254 (0.8)	1/293 (0.3)
Endocarditis	4/150 (2.7)	0/293 (0.0)

Values are mean±SD or n/N (%). COAPT indicates Clinical Outcomes Assessment of the MitraClip Percutaneous Therapy; HF, heart failure; MR, mitral regurgitation; NYHA, New York Heart Association; STS‐PROM, Society of Thoracic Surgeons Predicted Risk of Mortality; TMVR, transcatheter mitral valve replacement.

a Midterm follow‐up for TMVR cohort and COAPT device group were 10.1 and 12 mo, respectively.

b Unplanned mitral valve intervention within 24 mo.

Although both techniques have shown promising results, they have some potential theoretical advantages and limitations. TMVr may be technically more challenging, and mitral anatomy is not suitable in all patients. In addition, hemodynamic results with TMVr devices are less predictable, and the rates of at least moderate residual MR are ≈10%.^3^, ^4^ Safety and procedure‐related complications seem to favor TMVr, probably because of the widely used transfemoral approach, lower profile catheters, and consistent hemodynamic stability during the procedure. Moreover, some concerns have been raised about TMVR platforms. It is well known in the surgical field that in patients <65 years, bioprosthetic valves degenerate earlier, driving increased reoperation rates and shorter long‐term survival.^42^ However, to date, evidence regarding efficacy and long‐term durability with TMVR devices remain unclear. Furthermore, antithrombotic therapy in TMVR patients has yet to be formally established, and evidence is limited regarding the risk of valve thrombosis. Most platforms recommended a minimal anticoagulation treatment duration of 3 months postoperatively, usually based on current guidelines for surgical bioprosthetic valves. Further data are needed to determine the best antithrombotic strategy in this scenario. Consequently, both TMVR and TMVr systems should be considered as complementary treatments in patients with high surgical risk and severe MR.

### Study Limitations

This study has the limitations inherent to a systematic review that collects only information described in the publications or reported in international conferences; thus, relevant information might be omitted in the review that could shed some more light on this topic. In addition, this systematic review analyzed a limited series of patients for whom, in some cases, only collective data were available. Indeed, incomplete individual data regarding some patient baseline characteristics, procedure‐related aspects, and follow‐up may have prevented complete and accurate clinical evaluation. Finally, the reported patients might have had better outcomes than those who were not published (ie, selection bias).

In conclusion, TMVR has emerged as a less invasive alternative to conventional surgery for treatment of severe MR in patients with high or prohibitive surgical risk. The early experience with TMVR showed a high rate of technically successful valve implantations and excellent hemodynamic results with a low rate of residual MR. However, despite the heterogeneity of the results, procedure‐related complications and midterm mortality remained high. Improvement of patient selection, further iterations of TMVR systems, and a gradual increase in use of the transfemoral–transeptal approach might be necessary before the widespread adoption of this technology as an alternative to conventional surgery. The confirmation of TMVR as an option complementary to TMVr would drastically increase therapeutic options for patients with severe MR and increased surgical risk.

## Sources of Funding

Dr del Val is supported by a research grant from the Fundación Alfonso Martín Escudero (Spain). Dr Rodés‐Cabau holds the Research Chair “Fondation Famille Jacques Larivière” for the Development of Structural Heart Disease Interventions.

## Disclosures

Dr Rodés‐Cabau has received institutional research grants from Edwards Lifesciences and Medtronic. The remaining authors have no disclosures to report.
